# The unique genomic properties of sex-biased genes: Insights from avian microarray data

**DOI:** 10.1186/1471-2164-9-148

**Published:** 2008-03-31

**Authors:** Judith E Mank, Lina Hultin-Rosenberg, Matthew T Webster, Hans Ellegren

**Affiliations:** 1 Department of Evolutionary Biology, Evolutionary Biology Centre, Uppsala University, Norbyvägen 18D, SE 752 36 Uppsala, Sweden

## Abstract

**Background:**

In order to develop a framework for the analysis of sex-biased genes, we present a characterization of microarray data comparing male and female gene expression in 18 day chicken embryos for brain, gonad, and heart tissue.

**Results:**

From the 15982 significantly expressed coding regions that have been assigned to either the autosomes or the Z chromosome (12979 in brain, 13301 in gonad, and 12372 in heart), roughly 18% were significantly sex-biased in any one tissue, though only 4 gene targets were biased in all tissues. The gonad was the most sex-biased tissue, followed by the brain. Sex-biased autosomal genes tended to be expressed at lower levels and in fewer tissues than unbiased gene targets, and autosomal somatic sex-biased genes had more expression noise than similar unbiased genes. Sex-biased genes linked to the Z-chromosome showed reduced expression in females, but not in males, when compared to unbiased Z-linked genes, and sex-biased Z-linked genes were also expressed in fewer tissues than unbiased Z coding regions. Third position GC content, and codon usage bias showed some sex-biased effects, primarily for autosomal genes expressed in the gonad. Finally, there were several over-represented Gene Ontology terms in the sex-biased gene sets.

**Conclusion:**

On the whole, this analysis suggests that sex-biased genes have unique genomic and organismal properties that delineate them from genes that are expressed equally in males and females.

## Background

Many genes are more actively transcribed in one sex than the other, and this sex-biased expression pattern is a mechanism by which heritable sexual dimorphisms can arise from a genome that is largely identical in males and females [1]. Sex-biased gene expression is relatively common in metazoans [2-5], and has important evolutionary [1,6-9], medical [10-12], and genomic [13,14] implications.

Additionally, sex-biased genes are by their very nature often linked to reproduction, and this makes many sex-biased genes subject not only to natural selection, but the powerful pressures of sexual selection as well [15,16]. The reproductive role of sex-biased gene expression is most easily observed in the global transcription profiles of the gonad, which shows the highest degree of sex-biased gene expression of all organs [17,18]. In analysis where the entire animal was used for microarray analysis [2,7,19,20], the gonad is typically the highest contributor to sex-biased gene lists. However, somatic tissues can exhibit remarkably high sex-biased expression patterns as well [5,17], and these may produce secondary sexual characteristics and behaviors, or result from metabolic differences between males and females.

Microarray technologies have recently made it possible to study expression patterns for entire annotated transcriptomes, which has the potential to vastly increase our understanding of the underlying genomic mechanisms that give rise to recognizably dimorphic sexes. Initial evolutionary studies of transcriptome-wide sex-biased gene expression in *Drosophila melanogaster *suggested that sex-biased genes have peculiar evolutionary properties. Specifically, male-sex-biased genes, particularly those associated with gonad, evolve at a faster functional rate [9,21-23], have higher rates of recombination that are tied to higher GC levels [14,24], and are non-randomly distributed among the fly chromosomes [4,25].

Recent results have suggested that the syndrome of sex-biased genes observed in *D. melanogaster *may not extend to all metazoans. Work in other Drosophila species did not recover an elevated rate of evolution for male-biased genes [26], and recent work in birds indicated that female-biased genes exhibit the highest rate of functional change [6]. Birds are particularly of interest due to their female heterogametic (ZZ-ZW) system of sex chromosome inheritance, which allows for the analytical partitioning of maleness from heterogamety. Genomes with female heterogamety are subjected to different evolutionary forces than male heterogametic genomes [27], which can make birds a revealing contrast to male heterogametic mammalian and *Drosophila *lineages.

Elucidating underlying genomic and molecular differences between sex-biased and unbiased genes can give insight into the functionality and constraints for these expression classes, and ultimately help us understand the forces that produce their unique evolutionary properties. Global analysis of sex-biased gene expression in female heterogametic systems have so far been limited to birds [17,28] and frogs [29], and a detailed understanding of the genomic properties of sex-biased genes in a female heterogametic system is warrented in order to understand how this sex chromosome system can effect sex-specific evolutionary pathways.

Here we study several expression (tissue specificity, expression level variance, gene function) and genomic (chromosome distribution, GC content, codon bias) properties of gene expressed in somatic (brain and heart) and gonad tissue of embryonic chickens, and investigate how these properties relate to sex-biased genes.

## Results

### Expression properties

The genomic distribution of biased and unbiased genes is shown in Table 1, where sex-bias has been denoted by both fold-change and significance parameters. When comparing the percentage of genes that are sex biased from the total pool of expressed genes in a given tissue, roughly 18% of genes were significantly-biased at the absolute log_2 _fold-change level >1, though this proportion decreased as the fold-change level increased. Z-linked targets were consistently more sex-biased across a range of fold-change cutoffs compared to autosomal targets. Specifically, male-biased genes more frequent on the Z, most likely due to the lack of dosage compensation recently suggested for birds [17,28]. Female-biased genes were more common on the autosomes for both heart and gonad, with autosomal female-biased and male-biased genes being roughly equal for brain. The gonad showed the highest proportion of sex-biased genes.

**Table 1:** Distribution of microarray targets showing significant (*p *< 0.05) expression differences between male and female 18 day embryos in brain, heart, and gonad.

Tissue		Significantly Expressed Targets	Absolute log_2 _Fold Change	Female	Male	Total Biased Targets
Heart	Autosomes	11892	> 1	122 (1.0%)	44 (0.4%)	166 (1.4%)
			> 1.5	30 (0.3%)	7 (0.06%)	37 (0.4%)
			> 2	14 (0.1%)	2 (0.02%)	16 (0.3%)
	
	Z chromosome	480	> 1	7 (1.4%)	54 (11.3%)	61 (12.7%)
			> 1.5	4 (0.8%)	10 (2.1%)	14 (2.9%)
			> 2	4 (0.8%)	1 (0.2%)	5 (1.0%)

Brain	Autosomes	12454	> 1	55 (0.4%)	72 (0.6%)	127 (1%)
			> 1.5	16 (0.2%)	17 (0.1%)	33 (0.3%)
			> 2	8 (0.06%)	5 (0.04%)	13 (0.1%)
	
	Z chromosome	525	> 1	4 (0.8%)	78 (15%)	82 (15.8%)
			> 1.5	3 (0.6%)	4 (0.8%)	7 (1.4%)
			> 2	2 (0.4%)	0 (0.0%)	2 (0.4%)

Gonad	Autosomes	12746	> 1	1292 (10.1%)	936 (7.4%)	2228 (17.5%)
			> 1.5	751 (5.9%)	474 (3.7%)	1225 (9.6%)
			> 2	484 (3.8%)	251 (2.0%)	735 (5.8%)
	
	Z chromosome	555	> 1	27 (4.9%)	187 (33.7%)	214 (38.6%)
			> 1.5	20 (3.6%)	71 (12.8%)	91 (16.4%)
			> 2	12 (2.2%)	28 (5.0%)	40 (7.2%)

All tissues*	Autosomes	9124	> 1	3 (0.03%)	1 (0.01%)	4 (0.04%)
	
	Z	372	> 1	0 (0%)	15 (4.0%)	15 (4.0%)

* same direction

**Figure 1:**
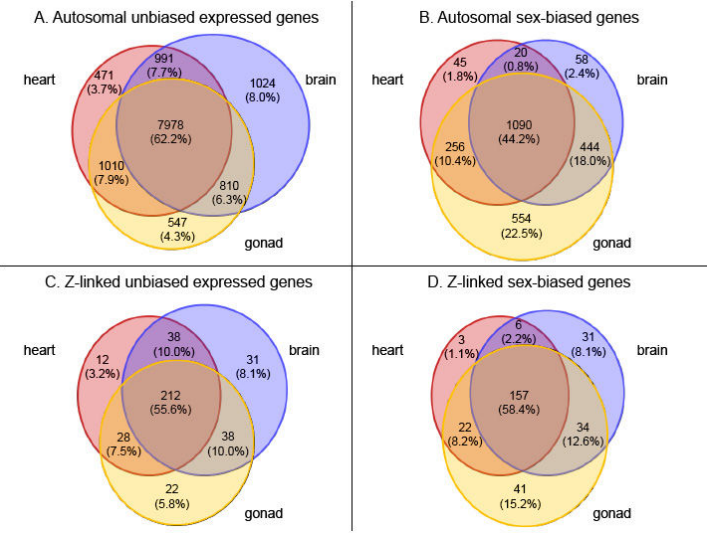
Venn diagram showing tissue specificity for unbiased and sex-biased genes for brain (blue), heart (red) and gonad (yellow). Panel A. Expression intersection of autosomal unbiased genes. Panel B. Expression intersection of autosomal sex-biased genes. Panel C. Expression intersection of Z-linked unbiased genes. Panel D. Expression intersection of Z-linked sex-biased genes. Sex-biased genes identified as significantly differentially expressed (*p*_*adj *_< 0.05, absolute log_2 _fold-change > 1) in at least one tissue analyzed.

**Figure 2:**
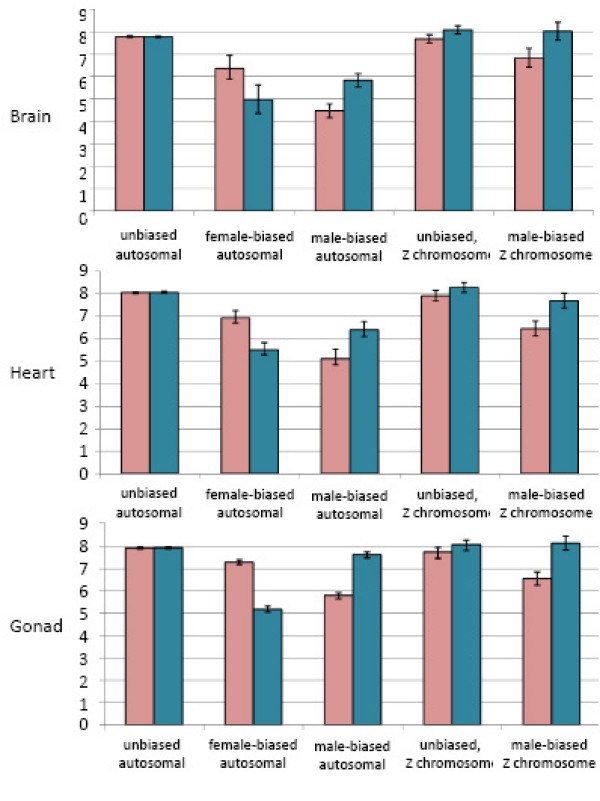
Average relative expression for unbiased, female-biased, and male-biased gene sets for the three tissues analyzed. Sex-biased was determined for each tissue separately, and genes that were differentially expressed between males and females (p_adj _< 0.05) with an absolute log_2 _fold-change > 1 were classed as sex-biased. Pink bars represent the average across female replicates, blue bars represent the average across male replicates. 95% confidence whiskers are shown, based on bootstrapping (1000 repetitions). Due to small sample sizes, the female-biased Z-linked category is not shown. See Table 1 for numbers of genes in each expression class. The Y-axis corresponds to relative expression levels.

There were several distinct expression patterns that delineate sex-biased genes from the remainder of the transcriptome. First, sex-biased genes were expressed in fewer tissues than unbiased genes for both autosomal and Z-linked microarray targets (*p *< 0.00001 for both Z and autosomal genes), as determined by equality of proportions test for overlapping regions (Fig. 1). Additionally, based on normalized florescence levels from the microarrays, sex-biased autosomal genes within each tissue were expressed at consistently lower levels than unbiased genes (Fig. 2), even for the sex with higher expression. Z-linked genes show a slightly different pattern however, as the average expression level across male replicates was statistically the same for Z-linked male-biased and unbiased genes. The average expression across female replicates for male-biased genes was somewhat lower than for unbiased genes.

**Figure 3:**
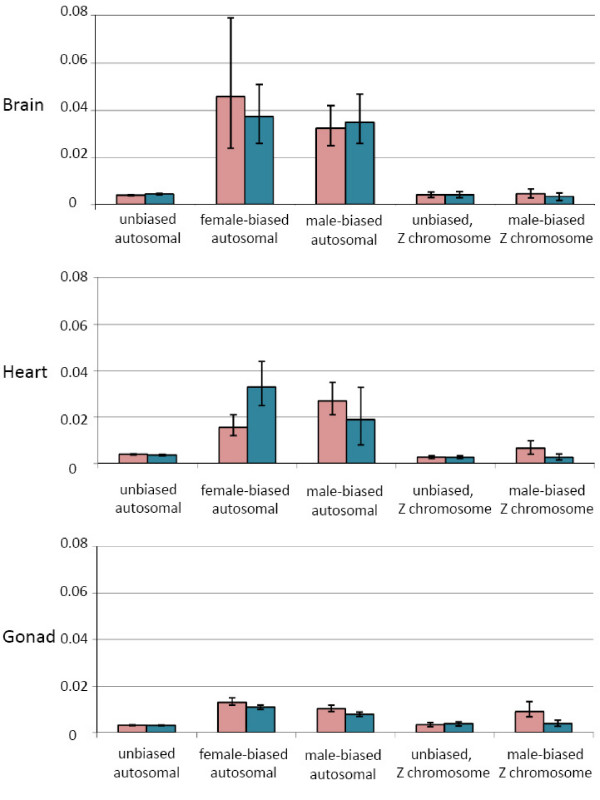
Average within-sex gene expression variance for sex-biased expression categories. Sex-biased was determined for each tissue separately, and genes that were differentially expressed between males and females (p_adj _< 0.05) with a absolute log_2 _fold-change > 1 were classed as sex-biased. Pink bars represent the average across female replicates, blue bars represent the average across male replicates. The Y-axis represents expression variance values. 95% confidence whiskers are shown, based on bootstrapping (1000 repetitions). Due to small sample sizes, the female-biased Z-linked category is not shown. See Table 1 for numbers of genes in each expression class.

Expression noise, or the variance in expression among individuals, has been shown to negatively correlate with gene essentiality, or those genes that are associated with the core functions in an organism [30-32]. We therefore calculated a normalized variance estimate for the expression among within-sex replicates as a proxy for expression noise. Sex-biased autosomal gene categories had higher within-sex variance values than unbiased autosomal genes in all three tissues (Fig. 3). There was far less difference in variance between Z-linked unbiased and male-biased gene sets.

### Clustering of genes with similar patterns of expression

Genes with similar breadth and level of expression have been shown to cluster in eukaryotic genomes [33-35], probably due to passive co-regulation of neighboring genes. We tested this possibility by calculating the average difference between both normalized level of expression between neighboring autosomal genes for which we had expression data, taking data from each tissue separately. We calculated the significance of local similarities by comparing these statistics with those derived from 10000 replicates where gene position was randomized on each chromosome. Neighboring genes showed significantly correlated levels of expression in gonad (*p *= 0.0002) and heart (*p *= 0.0114) although not in brain (*p *= 0.1848).

We also looked for clustering of sex-biased genes. Fold-change in expression, based only upon difference in expression between the sexes without a statistical cut-off p-value to define sex-bias, was significantly correlated in gonad (*p *< 0.0001), heart (*p *= 0.0175) and brain (*p *= 0.0131). All these correlations remained significant at the same level upon removal of comparisons of neighboring paralogs. This suggests that tandem duplications are not responsible for the observed local similarities, and that genes with similar expression patterns for some tissues are more likely than chance to be proximately located.

We also examined the genome for neighboring genes that both had significant evidence for biased expression in the same sex using the absolute value of log_2 _fold change > 1 with *p*_*adj *_< 0.05 to denote sex-bias. There were 157 such pairs using expression in gonad, compared with 100.9 expected by chance. When tandem duplicates were excluded, we observed 144 neighbor pairs, compared with 97.8 expected by chance alone. Both these associations are statistically significant (*p *< 0.0001 in both cases). However, when brain or heart expression was considered, there were no such examples of neighboring sex-biased genes.

### GC3 and codon bias

**Figure 4:**
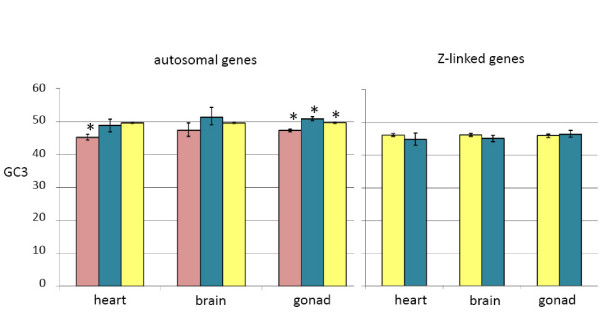
Average third position GC (GC3) content for expression categories. Sex-biased expression was determined with absolute log_2 _fold-change > 1 cutoff (*p*_*adj *_< 0.05). 95% confidence intervals (based on bootstrapping, 1000 repetitions) are shown. Sex-biased categories that differed significantly from unbiased genes in the same tissue are indicated (*, *p *< 0.05, see materials and methods for statistical metrics). Pink bars represent female GC3 values, blue bars represent male values, and yellow bars represent unbiased values. The Y-axis represents the proportion of coding sequence composed of G or C.

Third position GC content (GC3) has been linked to codon bias [36], which has in turn been demonstrated for different classes of sex-biased genes in *D. melanogaster *[14]. We found that GC3 differed by expression class for autosomal heart and gonad genes (one-way ANOVA, 2 d.f., *p *< 0.0001 in both cases). For the heart, female-biased genes had lower GC3 content than unbiased genes (Tukey's post hoc test, *p *< 0.01). GC3 for both male-and female-biased genes expressed in the gonad was significantly different than the unbiased gene class (*p *< 0.01 in both cases). There was no difference in GC3 among Z-linked expression classes (Fig. 4).

**Figure 5:**
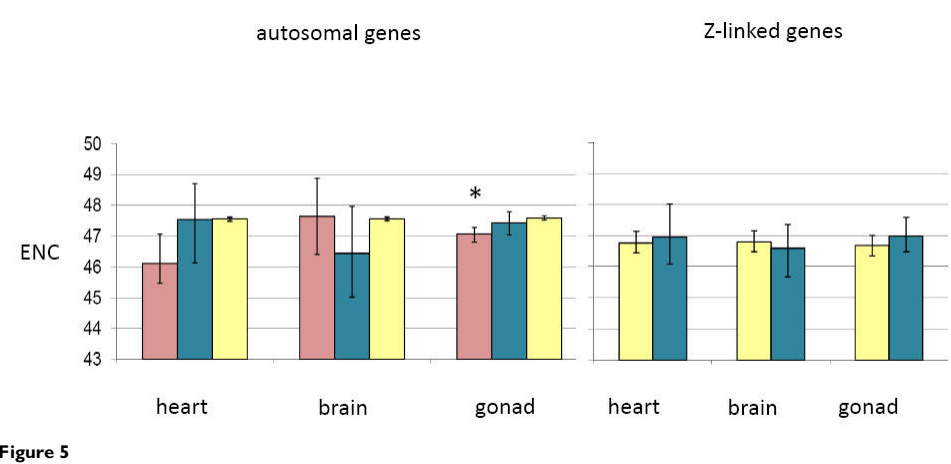
Average effective number of codons (ENC) for expression categories. Sex-biased expression was determined with absolute log_2 _fold-change > 1 cutoff (*p*_*adj *_< 0.05). 95% confidence intervals (based on bootstrapping, 1000 repetitions) are shown. Sex-biased categories that differed significantly from unbiased genes in the same tissue are indicated (*, *p *< 0.05, see materials and methods for statistical metrics). Pink bars represent female GC3 values, blue bars represent male values, and yellow bars represent unbiased values. ENC can theoretically range from 20 (extreme bias) to 61 (where all alternate codons are equally likely).

Codon usage, as measured by the effective number of codons (ENC) [37] also differed by expression class for autosomal heart and gonad genes (one-way ANOVA, 2 d.f., *p *< 0.003 in both cases), though not as starkly as GC3 levels (Fig. 5). Post hoc testing indicated that only autosomal female-biased genes expressed in the gonad showed more codon bias than genes with roughly equal expression in males and females.

**Figure 6:**
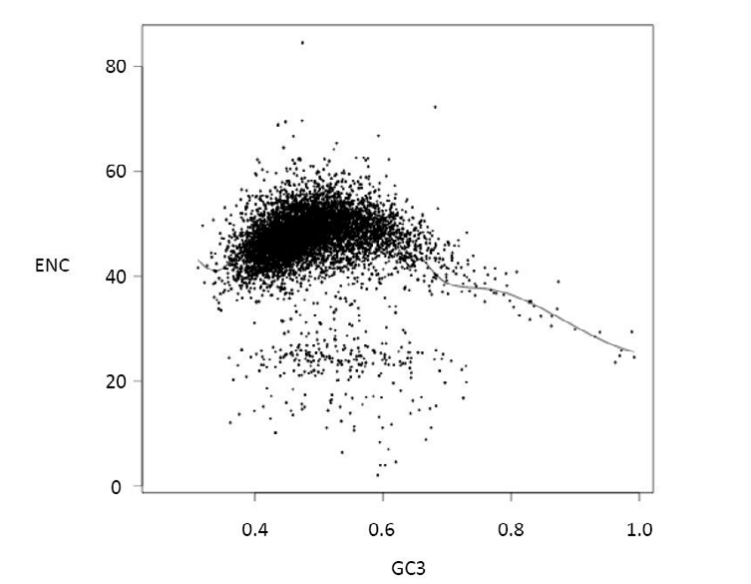
Relationship between GC3 and codon usage bias (ENC) in significantly expressed genes. A smoothed spline has been fitted to the data and is shown.

Figure 6 shows the relationship between GC3 and codon usage bias (ENC) in the genes in our dataset, with a smoothed spline fitted to the data. Codon bias is greatest when GC3 is skewed away from equal usage of GC or AT base pairs. The same relationship is found if the GC content of surrounding noncoding regions is used instead of GC3. When the ENC values of each sex bias category were compared with those predicted by GC3 from the model fitting, no significant differences were found (one-way ANOVA on residuals), suggesting that GC3 values do not predict codon-bias in our sex-biased expression categories.

### Gene Ontology

Many Gene Ontology (GO) terms were significantly over-represented (*p *< 0.01) in sex-biased categories within each tissue (Tables 2, 3, 4). The vast majority of terms were not significant after Bonferroni correction for multiple comparisons, though it is important to consider that this correction is excessively over-conservative. For heart expressed genes, *actin *binding and *sialyltranferase *activity were the most statistically over-represented terms for female-biased and male-biased autosomal gene sets respectively. For brain sex-biased genes, *S*-*methyltransferase activity *and *base excision repair *were the most strongly over-represented for female-and male-biased autosomal gene sets, though none of these over-representations were significant after Bonferroni correction. The only significant over-represented terms after multiple comparison correction were associated with the gonad, with *membrane associated *genes over-represented (*p*_*adj *_= 0.034) in male-biased gonad-expressed genes, and the *extracellular region *(*p*_*adj *_= 0.075) over-represented in female-biased gonad genes. Unbiased genes expressed in the gonad showed nineteen significant GO terms. This GO term dataset does not contain any gamete-specific terms, and this is likely due to the fact that the individual animals were collected at embryonic day 18, far before the onset of gametogenesis in chickens.

**Table 2:** Over-represented gene ontology terms for sex-biased categories (log_2 _fold-change. < 1,-> 1) within heart-expressed genes. Significantly (*p *< 0.01) over-represented terms are shown in term-for-term comparisons. Subsequent adjustments with the Bonferroni correction for multiple comparisons are shown.

Location	Sex-bias	GO	Name	Process	*p *(*p*_adj_)
Autosomal	Female	0003779	Actin binding	M	0.009 (1.00)
		0005515	Protein binding	M	0.01 (1.00)
	
	Male	0003373	Sialyltransferase activity	M	0.007 (0.92)
		0031228	Intrinsic to Golgi membrane	C	0.007 (0.92)

Z	Male	0009308	Amine metabolic process	B	0.009 (1.00)
		0006520	Amino acid metabolic process	B	0.009 (1.00)
		0006519	Amino acid and derivative process	B	0.009 (1.00)
		0006082	Organic acid metabolic process	B	0.009 (1.00)
		0019752	Carboxylic acid metabolic process	B	0.009 (1.00)
		0006807	Nitrogen compound metabolic process	B	0.009 (1.00)

**Table 3:** Over-represented gene ontology terms for sex-biased categories (log_2 _fold-change < 1,-> 1) within brain-expressed genes. Significantly (*p *< 0.01) over-represented terms are shown in term-for-term comparisons. Subsequent adjustments with the Bonferroni correction for multiple comparisons are shown.

Location	Sex-bias	GO	Name	Process	*p *(*p*_adj_)
Autosomal	Female	0008898	Homocysteine S_- _methyltransferase activity	M	0.004 (0.63)
		0008172	S-methyltransferase activity	M	0.004 (0.63)
		005891	Voltage-gated calcium channel complex	M	0.004 (0.63)
	
	Male	0006284	Base excision repair	B	0.008 (1.00)
		0004332	Fructose bisphosphate aldolase activity	M	0.008 (1.00)
		0016832	Aldehyde-lyase activity	M	0.008 (1.00)
		0030313	Cell envelope	C	0.008 (0.92)
		0030312	External encapsulating structure	C	0.008 (1.00)
		0044462	External encapsulating structure part	C	0.008 (1.00)
		0009279	Cell outer membrane	C	0.008 (1.00)

Z	Male	0008150	Biological process	B	0.009 (1.00)

**Table 4:** Over-represented gene ontology terms for sex-biased categories (log_2 _fold-change < 1,-> 1) within gonad-expressed genes. Significantly (*p *< 0.01) over-represented terms are shown in term-for-term comparisons. Subsequent adjustments with the Bonferroni correction for multiple comparisons are shown.

Location	Sex-bias	GO	Name	Process	*p *(*p*_adj_)
Autosomal	Female	005576	Extracellular region	C	0.000 (0.075)
		0005874	Microtubule	C	0.000 (0.21)
		0005509	Calcium ion binding	M	0.002 (0.24)
		0030705	Cytoskeletal-dependent intracellular transport	B	0.002 (0.99)
		0007018	Microtubule-based movement	B	0.002 (1.00)
		0007017	Microtubule-based process	B	0.003 (1.00)
		0015630	Microtubule cytoskeleton	C	0.003 (1.00)
		0044430	Cytockeletal part	C	0.004 (1.00)
		0030136	Clathrin-coated vesicle	C	0.005 (1.00)
		0048878	Chemical homeostasis	B	0.006 (1.00)
		0006828	Iron ion transport	B	0.006 (1.00)
		0050801	Ion homeostasis	B	0.006 (1.00)
		0030286	Dynein complex	C	0.006 (1.00)
		0030234	Enzyme regulator activity	M	0.01 (1.00)
	
	Male	0016020	Membrane	C	0.000(0.034)
		0005244	Voltage-gated ion channel activity	M	0.003 (1.000)
		0005249	Voltage-gated potassium channel activity	M	0.003 (1.000)
		0051536	Iron-sulfur cluster binding	M	0.003 (1.000)
		0051540	Metal cluster binding	M	0.003 (1.000)
		0004713	Protein-tyrosine kinase activity	M	0.004 (1.000)
		0009055	Electron carrier activity	M	0.007 (1.000)
		0016021	Integral to membrane	C	0.008 (1.000)
		0031224	Intrinsic to membrane	C	0.008 (1.000)
		0015020	Glucuronosyltransferase activity	M	0.008 (1.000)
		0015018	Galactosylgalactosylxylosylprotein 3-beta glucuronosyltransferase activity	M	0.008 (1.000)
		0007155	Cell adhesion	B	0.009 (1.000)
		0022610	Biological adhesion	B	0.009 (1.000)
		0044425	Membrane part	C	0.01 (1.000)

	Unbiased	0044237	Cellular metabolic process	B	0.000 (0.11)
		0044238	Primary metabolic process	B	0.000 (0.19)
		0016881	Acid-amino acid ligase activity	M	0.000 (0.42)
		005622	Ubiquitin cycle	C	0.000 (0.69)
		0006512	Intracellular	B	0.000 (0.70)
		0019787	Small conjugating protein ligase activity	M	0.001 (0.89)
		0008152	Metabolic process	M	0.001 (1.00)
		0016879	Ligase activity, forming carbon-nitrogen bonds	B	0.001 (1.00)
		0043170	Macromolecule metabolic process	B	0.001 (1.00)
		0016874	Ligase activity	M	0.001 (1.00)
		0005736	Mitochondrion	C	0.002 (0.15)
		0009056	Catabolic process		0.004 (0.24)
		0019538	Protein metabolic process		0.004 (1.00)
		0044248	Cellular catabolic process		0.005 (1.00)
		0003676	Nucleic acid binding		0.005 (1.00)
		0044429	Mitochondrial part		0.006 (1.00)
		0043283	Biopolymer metabolic process		0.006 (1.00)
		0006412	Translation		0.006 (1.00)
		0044267	Cellular protein metabolic process		0.007 (1.00)
		0043231	Intracellular membrane-bound organelle		0.008 (1.00)
		0043227	Membrane-bound organelle		0.008 (1.00)
		0044260	Cellular macromolecule metabolic process		0.008 (1.00)
		0006396	RNA processing		0.009 (1.00)

## Discussion

The data in this study was collected from both somatic and gonad tissue of embryonic day 18 chicks, after the circulating testosterone and estrogen levels diverge between males and females and initiate sexual differentiation [38]. This suggests that the sex-biased genes identified in this study are involved in development of sexually dimorphic anatomy, physiology, and behavior. However, gametogenesis does not commence until well after hatching in chicken, therefore these experiments are more focused on the development of the gonad than on genes specific to gametogenesis. It is therefore difficult to compare the degree of sex-bias observed here to studies of other organisms taken at different stages of the life-cycle [5,23,29]. Sex-biased gene expression will increase with the onset of sexual maturity, due both to the expression of gamete-specific transcripts and genes with sex hormone-receptors. However, in general it would be expected that juvenile individuals would show lower levels of sex-biased gene expression than adults in all tissues, and this is indeed what we observe. The degree of sex-bias for autosomal genes is less in our juvenile gonad tissue when compared both to *Xenopus laevis *[29] and *Drosophila melanogaster *[18] adult tissue at similar fold-change cut-off levels. Chicken embryonic brain tissue also showed less degree of sex-bias for autosomal genes than adult mouse brains [5] at similar fold-change levels.

### Expression properties

Sex-biased genes were present in both somatic and gonad tissues, though the gonad showed the highest proportion of sex-biased gene expression across the range of fold-change cutoff values. This likely stems from the fact that the gonad is the most sexually dimorphic organ, and although the testes and ovary share a common precursor, in many ways they represent two distinct tissue sets. It is therefore logical that sex-biased gene expression would be the most pronounced in this region.

Sex-biased genes share several expression properties that have implications to their evolution. First, sex-biased genes were more tissue specific in our analysis (Fig. 1), which is consistent with previous work suggesting sex-biased genes are more narrowly expressed than unbiased genes [5,39]. Coding regions that are broadly expressed face many, sometimes contradictory, evolutionary constraints on gene expression evolution [40], and it may be that genes with extensive expression throughout an organism are less able to evolve sex-biased expression patterns without disrupting critical functions in other tissues.

In addition to being expressed at lower levels (Fig. 2), sex-biased genes also showed more variance in expression (Fig. 3), at least for genes mapped to autosomal locations. Variance is an indicator of expression noise, and genes with larger amounts of expression noise have been shown to be less important in organismal survival and functionality [30-32]. Somatic organs are functionally identical in males and females, therefore sex-biased autosomal genes expressed in these tissues theoretically do not have important developmental or regulatory roles. The reduced difference between autosomal biased and unbiased genes in the gonad may reflect the sex-specific nature of the ovary and testes, where the pattern of sex-biased expression observed in the gonad is required for the formation of these distinct tissues. The lack of dosage compensation of the Z chromosome means that most genes have a default male-biased expression pattern, and this confounds the analysis of male-biased Z-genes and essentiality. The reduced variance observed across all tissues for male-biased Z-linked genes when compared to male-biased autosomal genes likely stems from the default male-biased expression pattern, as male-biased genes may have more important roles in the organism, but still exhibit male-biased expression simply due to Z-linkage. Overall, these findings suggest that somatic sex-biased genes, particularly those attached to the autosomes, may have less critical roles for organismal survival than unbiased genes.

There are indications [6] that avian sex-biased genes show an altered form of the accelerated rates of protein evolution seen in other animals[4,9], with female biased genes in birds having the highest rate of functional change. The above-described connections between tissue specificity, expression level, and expression variance and sex-bias may offer explanations for this rapid rate of evolution. Rates of evolution have been positively correlated with tissue specificity [41,42], and negatively correlated with overall expression level [43,44]. Additionally, increased variance in expression level may indicate that a gene is not critical [30], and therefore subject to less evolutionary constraint, which could theoretically manifest in an accelerated rate of protein change for genes with high expression variance. This would mean that genes which are tissue specific, have low expression levels, as well as high expression variances, are also likely to exhibit accelerated rates of protein evolution. Sex-biased genes possess all the characteristics, and it seems therefore likely that these characteristics play an important role in the rapid rate of evolution for sex-biased genes.

### Genomic distribution

We found significant clustering of similarly expressed genes. Neighboring genes in both the heart and gonad showed significant correlations in sex-averaged expression level. Additionally, when assessed just by fold-change, sex-biased genes in all three tissues showed significant clustering, though only gonad showed significant non-random distribution after adjusted-p-values were used to denote significantly sex-biased genes. All the observed clustering patterns, for both expression level and for sex-biased expression may best be explained by local effects such as bidirectional promoters and transcriptional read-through, which cause the transcription of genes to influence expression of their neighbors [45].

### GC3 and codon bias

Work in *D. melanogaster *has demonstrated different levels of codon-bias among sex-biased expression classes [14], and we recovered a similar pattern in chicken. Specifically, we observed codon-bias in female-biased genes expressed in the gonad. This could be explained be due to selection for specific codon usage patterns, or to differing GC3 levels that we observed across sex-biased categories (Fig. 4), as GC3 patterns can influence codon bias [36]. When we compared the codon-bias values of each sex bias category with those predicted by GC3 from the model fitting, we found no significant differences (one-way ANOVA on residuals), suggesting that there no evidence for any differences in selection for codon-usage between sex-biased and unbiased genes. Our observations therefore suggest that in chicken, as in mammals, codon-usage bias is mainly determined by the GC content of the isochore where each gene is situated, rather than natural selection acting on some aspect of gene expression [46].

### Gene Ontology

We found some over-representation of Gene Ontology terms in our sex-biased expression categories, particularly for those sex-biased genes expressed in the gonad. It is of course tempting to speculate about the possible reasons that over-represented GO terms are associated with sex-bias expression classes. While significantly over-represented GO terms from the male-and female-biased lists lack an immediately obvious sex-specific functionality, the unbiased gonad list is striking in the number of basal metabolic terminologies. Housekeeping and basal-functioning genes would not be expected to exhibit sex-biased expression, partly because they are important to both sexes and partly because they are generally broadly expressed and therefore less likely to exhibit sex-biased expression, as described above. This may explain the over-abundance of such terms as *cellular metabolic process, mitochondrion, and ligase activity*, which are theoretically common to all organs and tissues.

## Conclusion

We have presented a characterization of several properties of sex-biased genes identified from chicken microarray experiments. Sex-biased genes in chicken have a larger variance in expression level, are expressed in fewer tissues, and at lower overall levels than unbiased genes. Additionally, sex-biased genes have somewhat different GC3, and codon-bias properties. These qualities may help explain why sex-biased genes have unique evolutionary properties, and present a useful framework for future analyses of sex-biased expression patterns.

## Methods

### Sample collection and preparation

White Leghorn embryos were euthanized after 18 days of incubation (ed18), and the brain, heart, and left gonad collected from four male and four female individuals. This data was originally collected for an analysis of dosage compensation, and further details about study design, tissue preparation, RNA isolation, and microarray hybridization and analysis have been previously published [17]. We note only major experimental details and where the analysis presented here differs. All microarray data is available at the Gene Expression Omnibus (project GSE 8693).

### Annotation of probe sets

Annotations and genomic locations for the microarray probe sets were extracted from Ensembl [50] using version 2.1 of the chicken genome (WASHUC2 May 2006). All genes that were not assigned to a specific chromosome in this build were removed from further analysis, as many of these genes may be sex-linked and show dosage effects in expression. Additionally, as the W chromosome assembly is incomplete, the few W-assigned gene targets were also removed. The remaining dataset was comprised of coding regions that have been definitively mapped to either the autosomes or the Z chromosome. This dataset was then parsed into autosomal and Z components for all subsequent analyses, as the lack of effective and complete dosage compensation on the chicken Z chromosome [17,28] results in male-biased expression for Z-linked genes due to simple dosage effects, which confounds subsequent studies of expression bias.

### Identification of sex-biased genes

For each actively transcribed target, the log_2 _(average male/average female) fold-change value was calculated. This treatment results in negative values for genes that are female-biased, positive values for genes that are male-biased, and values approaching 0 for genes with similar expression in both sexes. Genes were further divided into fold-change cutoff categories, based on the Wilcoxon test statistic of the log_2 _values, and adjusted for a 5% false discovery rate using the Benjamini Hochberg correction. Therefore, absolute value of log_2 _fold change > 1 corresponds to a 2× difference in expression in one sex compared to the other, absolute value of log_2 _fold change > 1.5 represents roughly a 3× increase, and absolute value of log_2 _fold change > 2 corresponds to a 4× difference. Relative expression was calculated across within-sex replicates for sex-biased (*p*_*adj *_< 0.05, absolute fold-change >1) and unbiased gene classes for each tissue separately, and 95% confidence intervals were determined via bootstrapping (1000 repetitions).

In order to investigate the relationship between expression variance, an indicator of noise, and fold-change, we normalized the expression level for each gene target such that the averaged expression level was equal to one within each set of sex-specific replicates. This reduces the problem where genes with low expression levels will have associated small variance estimates, which could create a false signal. We then computed average within-sex variance for each sex-bias expression class (male biased log_2 _fold change > 1, *p*_*adj *_> 0.05; female biased genes where log_2 _fold change < 1- and *p*_*adj *_< 0.05; and unbiased probe sets), and computed 95% confidence intervals via bootstrapping (1000 replicates).

### Clustering of genes with similar patterns of expression

For all genes that were significantly expressed in our study, we calculated the difference in average level of expression between it and its nearest neighbor for which gene expression had been measured in the same tissue. We used sex-averaged expression to correct for sex-bias effects. We also measured the difference in fold-change between neighboring gene pairs for which we had expression data, and counted the number of times gene pairs both had evidence for significant biased expression in the same sex (absolute log_2 _fold change > 1, *p *< 0.05). We examined the significance of these values by comparing them with datasets where gene order was randomized on each chromosome using 10000 replicates.

### GC3 and bias in codon usage

Third codon GC (GC3) content has been linked to codon-bias [36,47], which has in turn been linked to sex-biased gene expression patterns in *Drosophila *[14]. Therefore, in order to investigate the relationship between sex-biased gene expression and codon bias, we calculated GC3 and the Effective Number of Codons (ENC) [37] from the Ensembl annotation of the chicken genome. ENC can range from 20, in the case of extreme bias where only one codon is used for each amino acid, to 61, where all alternative codons are equally likely. We calculated the average GC3 and ENC for each sex-bias expression category within each tissue at the fold-change < 1- or > 1, *p*_*adj *_< 0.05 cutoff. 95% confidence intervals were computed with bootstrapping (1000 replicates). Within each tissue, we compared the GC3 and ENC among autosomal expression categories using one-way ANOVA (2 d.f.), and between Z-linked categories using a one-tailed t-test assuming unequal variance. As ENC is strongly governed by GC content in vertebrates, we also tested whether differences in ENC values between categories were significantly different from that predicted by the observed differences in GC3. To do this we fitted a smoothed cubic spline to the relationship between ENC and GC3, and used this to estimate predicted ENC values from GC3. Significance was tested performing a one-way ANOVA on the residuals of model fitting.

### Gene Ontology analysis

We compared the functional role of biased and unbiased gene sets for each tissue using Ontologizer [48] and the *Gallus *Gene Ontology (GO) [49] database to identify over-represented GO categories. For each tissue, the actively expressed genes were used as the population gene set in term-for-term comparisons with each of the sex-biased expression categories as separate study sets (absolute fold change > 1, *p*_*adj *_< 0.05 cutoffs to denote sex-bias). Significant over-representation was determined for each term with Fisher's exact test, and the Bonferroni correction was used for multiple comparisons. As the Bonferroni method can be unnecessarily over-conservative, we report over-represented terms that are significant before Bonferroni correction as well.

## Authors' contributions

HE AND JEM conceived of the study. JEM, LH-R, and MW analyzed the data. All co-authors contributed to writing the manuscript.
